# An adiponectin-S1P autocrine axis protects skeletal muscle cells from palmitate-induced cell death

**DOI:** 10.1186/s12944-020-01332-5

**Published:** 2020-07-01

**Authors:** Amy Botta, Kazaros Elizbaryan, Parastoo Tashakorinia, Nhat Hung Lam, Gary Sweeney

**Affiliations:** Department of Biology, York University, Toronto, ON M3J 1P3 Canada

**Keywords:** Adiponectin, Sphingosine-1 phosphate, Skeletal muscle cells, Palmitate, ROS, Lipotoxicity, Cell death, AdipoRon

## Abstract

**Background:**

The prevalence of type 2 diabetes, obesity and their various comorbidities have continued to rise. In skeletal muscle lipotoxicity is well known to be a contributor to the development of insulin resistance. Here it was examined if the small molecule adiponectin receptor agonist AdipoRon mimicked the effect of adiponectin to attenuate palmitate induced reactive oxygen species (ROS) production and cell death in L6 skeletal muscle cells.

**Methods:**

L6 cells were treated ±0.1 mM PA, and ± AdipoRon, then assays analyzing reactive oxygen species (ROS) production and cell death, and intracellular and extracellular levels of sphingosine-1 phosphate (S1P) were conducted. To determine the mechanistic role of S1P gain (using exogenous S1P or using THI) or loss of function (using the SKI-II) were conducted.

**Results:**

Using both CellROX and DCFDA assays it was found that AdipoRon reduced palmitate-induced ROS production. Image-IT DEAD, MTT and LDH assays all indicated that AdipoRon reduced palmitate-induced cell death. Palmitate significantly increased intracellular accumulation of S1P, whereas in the presence of AdipoRon there was increased release of S1P from cells to extracellular medium. It was also observed that direct addition of extracellular S1P prevented palmitate-induced ROS production and cell death, indicating that S1P is acting in an autocrine manner. Pharmacological approaches to enhance or decrease S1P levels indicated that accumulation of intracellular S1P correlated with enhanced cell death.

**Conclusion:**

This data indicates that increased extracellular levels of S1P in response to adiponectin receptor activation can activate S1P receptor-mediated signaling to attenuate lipotoxic cell death. Taken together these findings represent a possible novel mechanism for the protective action of adiponectin.

## Background

The prevalence of type 2 diabetes and obesity has continued to rise and with this various comorbidities have become pervasive [1]. Skeletal muscle is a metabolically active tissue in which there are high levels of mitochondria [2, 3]. Lipotoxicity in skeletal muscle is well recognized as a contributor to the development of insulin resistance [4, 5]. In addition, under lipotoxic conditions reactive oxygen species (ROS) are produced, leading to increased lipid peroxidation, which in turn also leads to increased levels of cell death [6, 7]. It has been shown that addition of palmitate to skeletal muscle cells leads to increased levels of superoxide [8, 9]. Previous research has shown that palmitate (PA) induced cell death is in part due to increased accumulation of distinct ceramide species [10, 11]. While increased levels of ceramide are considered to be toxic, several metabolites of ceramide, such as sphingosine-1 phosphate (S1P) are known to mediate beneficial cellular responses, such as anti-apoptotic effects [12].

Lack of adiponectin action has now been implicated in many disease states, perhaps most especially type 2 diabetes in obesity [13, 14]. Healthy individuals typically have high concentrations of adiponectin within the circulation [15]. In both diabetes and obesity circulating levels of adiponectin are significantly reduced [16–18]. Although the bulk of circulating adiponectin derives from adipose tissue, previous research has in fact shown that skeletal muscle can produce and secrete adiponectin [19, 20]. Adiponectin has been shown to regulate fatty acid metabolism in muscle and can lead to increased fatty acid uptake and decreased fatty acid synthesis [21, 22]. Previous research has shown that under high-fat feeding conditions in mice, adiponectin can improve insulin sensitivity and prevent damage to skeletal muscle cells [23]. Since lower levels of adiponectin have been implicated in several metabolic disease states [24–26] there has been significant interest in the identification of small molecule adiponectin receptor agonists. One such small molecule is AdipoRon, which has been shown to mimic adiponectin signaling both in multiple cell types and in animal models [27, 28].

One important feature of adiponectin action, which could be beneficial during lipotoxic conditions, is that it stimulates the production of S1P, a signaling sphingolipid that is formed from the conversion of ceramide into sphingosine by ceramidases and subsequent conversion of sphingosine into S1P by sphingosine kinase [29, 30]. Adiponectin receptor 1 (AdipoR1) and 2 (AdipoR2), both have intrinsic ceramidase activity which is activated after binding with adiponectin or receptor agonists [31, 32]. This increase in ceramidase activity leads to increased conversion of ceramide into S1P, thereby reducing the buildup of ceramides and related lipotoxic molecules [31–34]. Previous research has shown that S1P plays an important role in skeletal muscle regeneration [35, 36]. Additionally, increased levels of S1P have been linked to decreased insulin resistance and cell death [37–39]. However, whether adiponectin-mediated increases in S1P action are required to confer anti-lipotoxic effects in skeletal muscle cells has not been fully elucidated.

Here rat L6 skeletal muscle cells treated with palmitate with or without AdipoRon were used to determine intracellular and extracellular S1P levels, ROS production and cell death. A subset of cells were also treated cells with S1P and pharmacological inhibitors to enhance or reduce S1P production. The findings provide new knowledge on the mechanistic role of S1P in mediating beneficial effects of adiponectin in skeletal muscle cells.

## Methods

### Cell culture

Rat L6 skeletal muscle myoblasts were incubated in alpha modified Eagle medium (α-MEM, Wisent Inc., Saint-Jean-Baptist, Quebec, Canada) supplemented with 10% volume/volume (v/v) fetal bovine serum and 1% antibiotic/antimycotic solution (v/v, Wisent Inc., Saint-Jean-Baptist, Quebec, Canada). Prior to passage and seeding for experiments cells were grown to a maximum of 80% confluency in 75cm^2^ flasks at 37 °C and 5% CO2. L6 cells were plated and left overnight and then were incubated for 4 h with 0.5% BSA containing medium. Then 0.1 mM of PA dissolved in 3% bovine serum albumin (BSA) or BSA control was added. A subset of cells was treated with either 35 μM AdipoRon (Cayman Chemical, Ann Arbor, Michigan, United States), 2.5 μM sphingosine-1-phosphate (S1P, Sigma Aldrich, St. Louis, Missouri, United States), 5 μM sphingosine kinase inhibitor II (SKI-II, Sigma Aldrich, St. Louis, Missouri, United States), or 5 μM 2-Acetyl-5-tetrahydroxybutyl imidazole (THI, Sigma Aldrich, St. Louis, Missouri, United States) as indicated.

### Analysis of intracellular ROS

CellROX Green (Thermofisher Scientific, Waltham, Massachusetts, United States) was utilized to detect ROS production in live cells following manufacturers instructions using an EVOS FL Auto 2 Cell Imaging System (Thermofisher Scientific, Waltham, Massachusetts, United States). Additionally, for plate-based assays, 2′,7′-Dichlorofluorescin Diacetate (DCF-DA, Sigma Aldrich, St. Louis, Missouri, United States) was utilized to detect ROS as previously described [40].

### Assays to measure cell death

Image-IT DEAD Green Viability Stain (Thermofisher Scientific, Waltham, Massachusetts, United States) was utilized to detect cell death after 24 h incubation following manufacturers instructions using a Nikon ECLIPSE Ti2 (Nikon, Tokyo, Japan). Using a kit, the release of lactate dehydrogenase (LDH) was determined (G-Biosciences, St. Louis, Missouri, United States). Briefly, 25 ul of media was transferred to a separate 96-well plate. 25 ul of reconstituted substrate mix was then added and the plate was incubated for 30 min. Following which, 25 ul of stop solution was added and the absorbance was then measured at 490 nm. The 3-(4,5-dimethylthiazol-2-yl)-2,5-diphenyltetrazolium bromide (MTT) assay (Sigma Aldrich, St. Louis, Missouri, United States) was conducted by incubating cells with 5 mg/ml MTT in PBS for 1 h at 37 °C and 5% CO_2_. After incubation cells were washed with PBS. DMSO (Sigma Aldrich, St. Louis, Missouri, United States) was then added to dissolve the precipitated formazan. The absorbance was then measured at 570 nm.

### Measurement of S1P levels

L6 cells were plated in 6 well plates and were treated with either PA or BSA with or without the addition of 35 uM AdipoRon. After 24 h the media (1 ml) was removed from the cells and placed in a separate tube. The media was then concentrated to approximately 1/5th it’s initial volume. The cells were lysed with a 0.5% solution of Sodium dodecyl sulfate (SDS, Sigma Aldrich, St. Louis, Missouri, United States). Both media and cell lysates were then frozen until use. To determine the levels of S1P, an ELISA kit was utilized (MyBioSource, San Diego, California, United States).

### Statistical analysis

For CellROX Green experiments data, where no statistical analysis was conducted data is shown as mean ± 95% confidence intervals for 150 cells. For all other experiments data is shown as boxplots [41]. All data were analyzed using Mann-Whitney U test with differences being considered statistically significant at *P* < 0.05. Statistical analysis was conducted using GraphPad Prism 6 (GraphPad Software, San Diego, California, United States). As multiple comparisons were utilized the false discovery rate (FDR) was determined by correcting the obtained *P* values using the Benjamini-Hochberg procedure for multiple comparisons [42]. In instances where the *P* value is rendered nonsignificant due to the FDR calculation, this is indicated within the figure and the specific values are given within the results section.

## Results

### AdipoRon reverses palmitate-induced ROS production

**Fig. 1:**
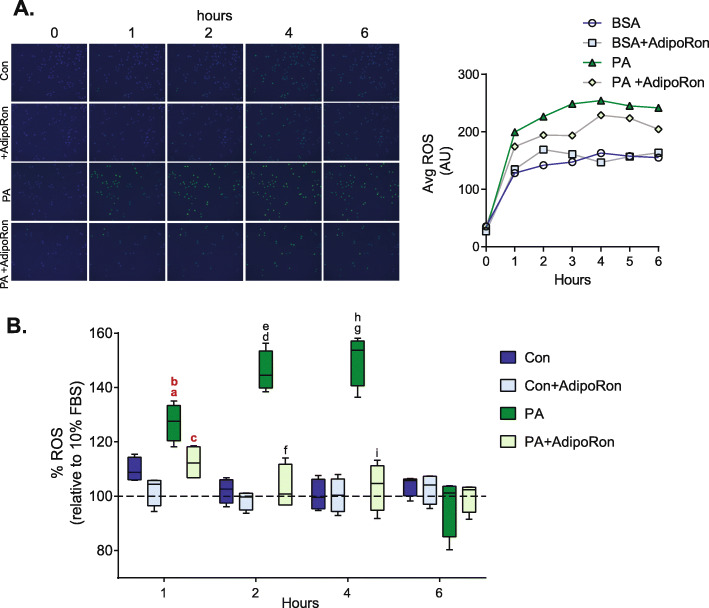
*AdipoRon attenuates palmitate induced ROS production.* L6 cells were incubated with 0.1 mM palmitate (PA) or vehicle control (Con) and were also treated with 35 μM AdipoRon as indicated for various timepoints as indicated following which ROS was measured using **a**) CellROX green live cell imaging (shown as mean ± 95% confidence intervals for 150 cells) or **b**) using DCF-DA. Graphs are displayed as boxplots; a = *P* < 0.05 vs 1 h Con, b = *P* < 0.05 vs 1 h Con+AdipoRon, c = *P* < 0.05 vs 1 h PA, d = *P* < 0.05 vs 2 h Con, e = *P* < 0.05 vs 2 h Con+AdipoRon, f = *P* < 0.05 vs 2 h PA, g = *P* < 0.05 vs 4 h Con, h = *P* < 0.05 vs 4 h Con+AdipoRon, i = *P* < 0.05 vs 4 h PA, red bolded letters indicate *P* values which were rendered nonsignificant > 0.05 after FDR calculation, *n* = 4–5

To determine the impact of incubation of PA and AdipoRon on ROS production in L6 cells a 12 h time course was conducted. ROS production was higher in PA-treated cells from 1 to 6 h (Fig. 1a). Coincubation of AdipoRon and PA reduced ROS production compared to PA treated cells. In contrast coincubation of control cells with AdipoRon did not alter levels of ROS production (Fig. 1a). To verify the findings of the live cell time course experiment, ROS production was measured using a DCF-DA plate based assay after 1, 2, 4, and 6 h of incubation with palmitate. While there was significance after 1 h of incubation for PA vs control (*P* = 0.03, FDR = 0.06), PA vs control + AdiponRon (*P* = 0.03, FDR = 0.06), and PA vs PA+ AdipoRon (*P* = 0.03, FDR = 0.06) after FDR calculation the corrected *P* value was greater than 0.05, indicating no significance. Similar to the results obtained from live cell imaging, incubation with palmitate significantly increased ROS production after 2 and 4 of incubation in comparison to BSA control. However, there was not a significant increase in ROS production after 6 h of incubation (Fig. 1b). To determine the impact of Adiponectin on PA-induced ROS production, a subset of cells were incubated with the adiponectin receptor agonist AdipoRon. Similar to the results obtained from live cell imaging incubation with AdipoRon significantly reduced PA-induced ROS production after 1, 2, and 4 h of incubation. No reduction in ROS production was observed after 6 h of incubation (Fig. 1b).

### AdipoRon reverses palmitate-induced cell death

**Fig. 2:**
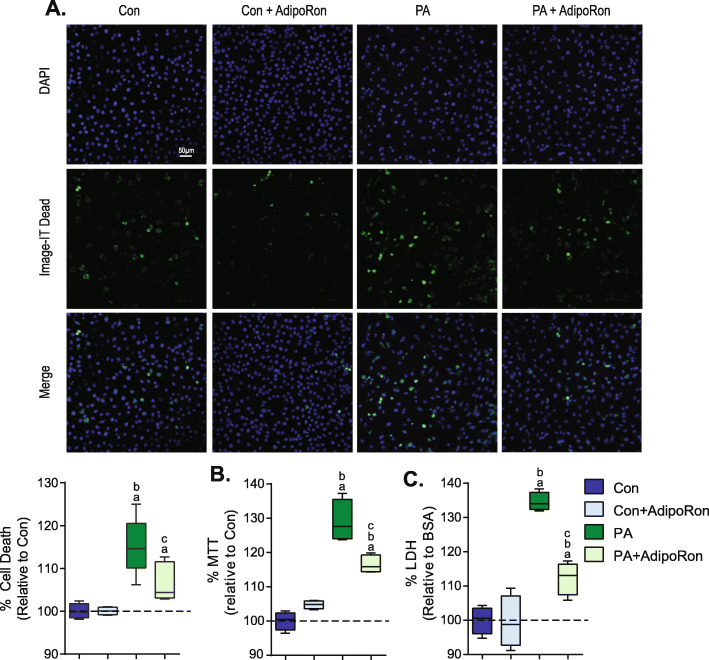
*AdipoRon attenuates palmitate induced cell death.* L6 cells were incubated with 0.1 mM palmitate (PA) or vehicle control (Con) and were also treated with 35 μM AdipoRon as indicated for 24 h after which cell death (shown as % toxicity) was measured using **a**) Image-IT DEAD Green Viability Stain **b**) MTT or **c**) LDH. Graphs are displayed as boxplots; a = *P* < 0.05 vs corresponding Con, b = *P* < 0.05 vs corresponding Con+AdipoRon, c = *P* < 0.05 vs corresponding PA n = 4

To determine the impact of PA and AdipoRon on cell death, cells were incubated with PA for 24 h with or without the addition of AdipoRon. Imaging with Image-IT Dead (Thermofisher Scientific) indicated that PA significantly increased cell death in comparison to controls (p. This increase in cell death was reversed by the coincubation of AdipoRon and PA (Fig. 2a). To verify these findings, MTT and LDH plate based assays were conducted. Similar to the microscopy results incubation with PA significantly increased cell death in comparison to BSA control. Coincubation with AdipoRon significantly reduced cell death in PA-treated cells. Coincubation with AdipoRon in BSA control cells did not significantly alter the level of cell death (Fig. 2b-c).

### AdipoRon significantly increases extracellular levels of S1P

**Fig. 3:**
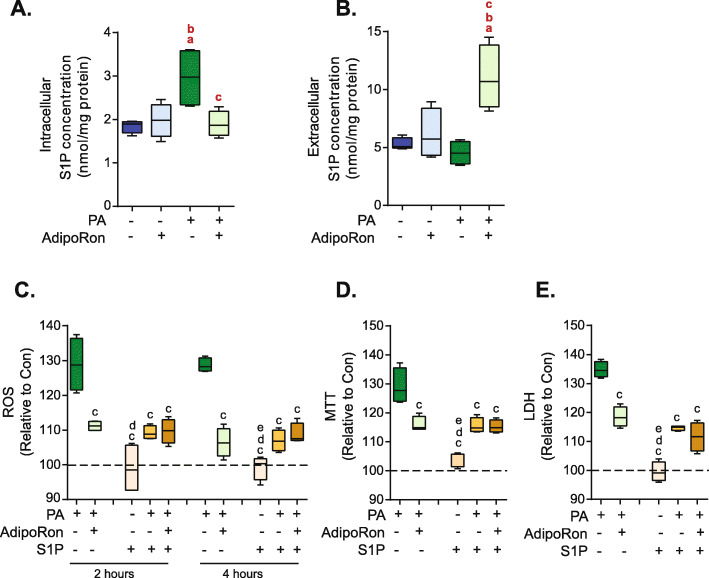
*AdipoRon increases the release of S1P from cells.* L6 cells were incubated with 0.1 mM palmitate (PA) or vehicle control (Con) and were also treated with 35 μM AdipoRon as indicated for 24 h after which S1P levels were measured in **a**) L6 cells and **b**) cell media. a = *P* < 0.05 vs Con, b = *P* < 0.05 vs Con+AdipoRon, c = *P* < 0.05 vs PA, red bolded letters indicate *P* values which were rendered nonsignificant > 0.05 after FDR calculation, *n* = 4. In a subset of cells 2.5 μM S1P was added for either 2 or 4 h following which **c**) ROS was measured using DCF-DA. Additionally after 24 h cell death (shown as % toxicity) was measured using **d**) MTT E) LDH. Graphs are displayed as boxplots; c = *P* < 0.05 vs PA, d = *P* < 0.05 vs PA + AdipoRon, e = *P* < 0.05 vs corresponding PA + S1P, n = 4

As palmitate can be used for the de novo synthesis of ceramide, which can subsequently be converted into S1P, the impact of incubation with palmitate on intracellular and extracellular concentrations of S1P was determined. Therefore, the concentration of S1P both within L6 cells (intracellular) and in the media (extracellular) after incubation with palmitate was determined. It was found that incubation with palmitate significantly increased intracellular levels of S1P (*P* = 0.03, FDR = 0.06) (Fig. 3a), however, incubation with palmitate did not cause a significant increase in extracellular levels of S1P (*P* = 0.03, FDR = 0.06) (Fig. 3b). In contrast, the addition of AdipoRon significantly decreased intracellular levels of S1P and significantly increased extracellular levels of S1P (*P* = 0.03, FDR = 0.06) (Fig. 3a-b). Addition of exogenous S1P significantly reduced PA-induced ROS production. Similarly, the addition of AdipoRon also significantly reduced PA-induced ROS production. However, coincubation of both S1P and AdipoRon did not lead to a further decrease in ROS production, therefore the effects of S1P and AdipoRon are not additive (Fig. 3c). Similar to ROS production incubation with either AdipoRon or S1P led to a significant decrease in PA-induced cell death as measured by MTT (Fig. 3d) and LDH (Fig. 3e). However, coincubation with both AdipoRon and S1P did not lead to a further decrease in cell death (Fig. 3d-e).

### Pharmacological manipulation of S1P significantly alters palmitate-induced ROS production and cell death

**Fig. 4:**
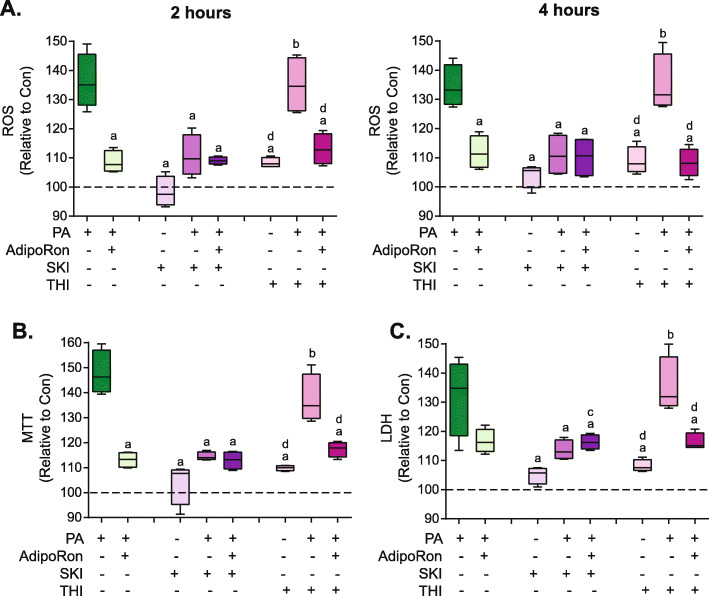
*Pharmacological manipulation of S1P significantly alters palmitate-induced ROS production and cell death.* L6 cells were incubated with 0.1 mM palmitate (PA) or vehicle control (Con) and were also treated with either 35 μM AdipoRon, 5 μM SKI-II, or 5 μM THI for either 2 or 4 h following which **a**) ROS was measured using DCF-DA. Additionally after 24 h cell death (shown as % toxicity) was measured using **b**) MTT or C) LDH. Graphs are displayed as boxplots; a = *P* < 0.05 vs corresponding PA, b = *P* < 0.05 vs corresponding PA + AdipoRon, c = *P* < 0.05 vs corresponding PA + SKI-II, d = *P* < 0.05 vs corresponding PA + THI, n = 4

To further investigate the role of S1P in the modulation of PA-induced ROS production and cell death sphingosine kinase inhibitor SKI-II which prevents S1P production was used. Incubation with SKI-II in control BSA cells did not increase ROS production in comparison to BSA alone. Incubation with SKI-II in PA-treated cells led to a significant decrease in PA-induced ROS production after 2 h and 4 h of incubation. However, the addition of AdipoRon did not lead to a further reduction in ROS production at either time point (Fig. 4a). In contrast to SKI-II which prevents S1P production, 2-Acetyl-4-tetrahydroxybutyl Imidazole (THI) inhibits S1P-lyase which catalyzes the irreversible decomposition of S1P to phosphoethanolamine and trans-2-hexadecenal, therefore THI increases levels of S1P. Incubation with THI in BSA treated cells significantly increased ROS production in comparison to BSA alone. Incubation of PA-treated cells with THI did not alter PA-induced ROS production after either 2 h or 4 h (Fig. 4a). Interestingly, coincubation of THI with AdipoRon significantly reduced PA-induced ROS levels. With respect to cell death incubation of PA treated cells with SKI-II significantly decreased cell death. Coincubation with AdipoRon did not lead to a further reduction in cell death (Fig. 4b-c). In contrast incubation with THI did not significantly reduce PA-induced cell death. However, coincubation with AdipoRon with THI produced a significant reduction in cell death (Fig. 4b-c).

## Discussion

Previous literature has shown the importance of skeletal muscle lipotoxicity in the development of various cellular consequences including insulin resistance [4, 43, 44], mitochondrial dysfunction [45, 46] and cell death [11, 47]. One feature of lipotoxic conditions is an increase in ROS production which can lead to cellular damage and eventually death [46, 48]. Both adiponectin signaling [23, 49–51], and more specifically S1P signaling [38, 52, 53], have been shown to confer anti-apoptotic effects in various cells and tissues. Several studies have now shown that S1P is an important mediator of many of the beneficial effects of adiponectin [32, 40]. However, the cross talk between these pathways in skeletal muscle under lipotoxic conditions which lead to cell death requires further investigation.

It was found that incubation with palmitate significantly increased ROS production after 1 h of incubation, with a maximal response being observed after 4 h of incubation. To determine ROS production CellROX green which reacts with superoxide [54], and DCF-DA which reacts with hydrogen peroxide and other reactive oxygen species [55]. This difference in specificity could explain why ROS signal was observed after 4 h but not after 6 h in the DCF-DA assay. Using palmitate to induce lipotoxic conditions, it was observed that adiponectin significantly reduced both ROS production and cell death in response to palmitate. This is consistent with conclusions from previous studies in endothelial cells and H9c2 cells [40, 56]. Furthermore, a separate study in endothelial cells found that addition of adiponectin reduced palmitate induced insulin resistance and inflammation, also via reduction of ROS production [57]. Previous studies in C2C12 myotubes and pancreatic β cells have also shown that palmitate increased production of S1P [52, 58], and a similar observation was made in this study. AdipoRon, an adiponectin memetic, is able to interact with both AdipoR1 and AdipoR2, which contain intrinsic ceramidase activity and lead to generation of S1P [31–33]. Importantly, it was also demonstrated that AdipoRon significantly increased extracellular levels of S1P. Hence, although both palmitate and AdipoRon act to increase S1P generation, S1P remains in the cell after incubation with palmitate alone. It is only in the presence of an input from AdipoRon-mediated signaling that extrusion of S1P to the extracellular media is facilitated. It was hypothesized that this S1P mediated beneficial autocrine effects, whereas accumulation of intracellular S1P was deleterious.

To directly study the potential importance of autocrine effects of S1P in a model of lipotoxic cell death, S1P was added directly to the cell culture medium to stimulate S1P receptors. Extracellular S1P significantly reduced palmitate-induced ROS production and cell death. Co-incubation of both S1P and AdipoRon did not produce an additive effect, suggesting that their respective effects were occurring through the same mechanism. Furthermore, the intrinsic regulation of S1P levels was modulated through the use of two pharmacological inhibitors: THI, an S1P-lyase inhibitor to increase intracellular S1P levels, and SKI-II, an S1P kinase inhibitor to reduce intracellular levels of S1P [59, 60]. In contrast to the addition of extracellular S1P, using THI to increase intracellular levels of S1P did not significantly reduce PA-induced ROS production or cell death and instead slightly increased. This is in keeping with previous studies which have shown that increased levels of S1P can lead to the increased production of a cytotoxic S1P metabolite trans-2-hexadecenal [61, 62]. Interestingly, coincubation of THI and AdipoRon caused enhanced release of S1P from the cell, allowing it to then function as a signaling molecule, and significantly reduced both ROS and cell death. However, future studies, such as incubation with PA, S1P inhibitors and AdipoRon coupled with inactivation of S1P receptors is necessary in order to determine the specific role of AdipRon and S1P receptors in mediating the palmitate induced cell death response.

## Conclusions

In summary, using an in vitro skeletal muscle myoblast model, it was shown that regulation of S1P generation, breakdown and secretion collectively play an important role in determining the consequences of palmitate-induced lipotoxicity. Although it is well established that adiponectin-signaling enhances ceramidase activity to generate S1P, a critical mechanism by which adiponectin exerts is beneficial anti-apoptotic effects is via also enhancing release of S1P which then exerts autocrine signaling effects. As adiponectin is a clinically relevant biomarker, with previous studies showing that high levels of circulating adiponectin correspond to lower cardiometabolic risk [63, 64]. Recently adiponectin has further been shown to correlate with the development of ischemic heart disease in normal glucose tolerance patients [65]. The findings presented in this study represent a possible novel mechanism for why higher levels of adiponectin are protective against the development and progression of metabolic diseases. Further studies are necessary in order to ascertain the clinical relevance of S1P in the development of cardiometabolic diseases.

## Data Availability

All data used to support the findings of this study are included in the article.

## Abbreviations

α-MEM: minimum Essential Medium Eagle - alpha modification
AdipoR1: adiponectin receptor 1
AdipoR2: adiponectin receptor 2
DCFDA: 2′,7′-Dichlorofluorescin diacetate
DMSO: dimethylsulfoxide
ELISA: enzyme-linked immunosorbent assay
FDR: false discovery rate
LDH: lactate dehydrogenase
MTT: (3-(4,5-dimethylthiazol-2-yl)-2,5-diphenyltetrazolium bromide)
PA: palmitate
PBS: phosphate buffered saline
ROS: reactive oxygen species
S1P: sphingosine-1 phosphate
SDS: sodium dodecyl sulfate
SKI-II: sphingosine kinase inhibitor II
THI: 5-Acetyl-5-tetrahydroxybutyl imadazole
v/v: volume/volume

## References

[1] Bhupathiraju SN, Hu FB (2016). Epidemiology of obesity and diabetes and their cardiovascular complications. Circ Res.

[2] Aon MA, Bhatt N, Cortassa SC (2014). Mitochondrial and cellular mechanisms for managing lipid excess. Front Physiol.

[3] Schrauwen P, Schrauwen-Hinderling V, Hoeks J, Hesselink MK (2010). Mitochondrial dysfunction and lipotoxicity. Biochimica et Biophysica Acta (BBA)-molecular and cell biology of. Lipids..

[4] Brøns C, Grunnet LG (2017). Mechanisms in endocrinology: skeletal muscle lipotoxicity in insulin resistance and type 2 diabetes: a causal mechanism or an innocent bystander?. Eur J Endocrinol.

[5] Badin P-M, Vila IK, Louche K, Mairal A, Marques M-A, Bourlier V (2013). High-fat diet-mediated lipotoxicity and insulin resistance is related to impaired lipase expression in mouse skeletal muscle. Endocrinology..

[6] Hauck AK, Bernlohr DA (2016). Oxidative stress and lipotoxicity. J Lipid Res.

[7] Lee H, Lim J-Y, Choi S-J. Oleate prevents Palmitate-induced atrophy via modulation of mitochondrial ROS production in skeletal Myotubes. Oxidative Med Cell Longev. 2017;2017. Article ID: 2739721. https://www.hindawi.com/journals/omcl/2017/2739721/. 10.1155/2017/2739721 PMC5602654 28947926

[8] Lambertucci RH, Hirabara SM, LdR S, Levada-Pires AC, Curi R, Pithon-Curi TC (2008). Palmitate increases superoxide production through mitochondrial electron transport chain and NADPH oxidase activity in skeletal muscle cells. J Cell Physiol.

[9] Yuzefovych LV, Solodushko VA, Wilson GL, Rachek LI (2012). Protection from Palmitate-induced mitochondrial DNA damage prevents from mitochondrial oxidative stress, mitochondrial dysfunction, apoptosis, and impaired insulin signaling in rat L6 skeletal muscle cells. Endocrinology..

[10] Dai Ly L, Xu S, Choi S-K, Ha C-M, Thoudam T, Cha S-K (2018). Oxidative stress and calcium dysregulation by palmitate in type 2 diabetes. Exp Mol Med.

[11] Turpin SM, Ryall JG, Southgate R, Darby I, Hevener AL, Febbraio MA (2009). Examination of ‘lipotoxicity’ in skeletal muscle of high-fat fed and Ob/Ob mice. J Physiol.

[12] Rutherford C, Childs S, Ohotski J, McGlynn L, Riddick M, MacFarlane S (2013). Regulation of cell survival by sphingosine-1-phosphate receptor S1P1 via reciprocal ERK-dependent suppression of Bim and PI-3-kinase/protein kinase C-mediated upregulation of Mcl-1. Cell Death Dis..

[13] Achari AE, Jain SK (2017). Adiponectin, a therapeutic target for obesity, diabetes, and endothelial dysfunction. Int J Mol Sci.

[14] Nigro E, Scudiero O, Monaco ML, Palmieri A, Mazzarella G, Costagliola C (2014). New insight into adiponectin role in obesity and obesity-related diseases. BioMed Res Int.

[15] Kizer JR (2014). Adiponectin, cardiovascular disease, and mortality: parsing the dual prognostic implications of a complex adipokine. Metab Clin Exp.

[16] Seino Y, Hirose H, Saito I, Itoh H (2007). High molecular weight multimer form of adiponectin as a useful marker to evaluate insulin resistance and metabolic syndrome in Japanese men. Metabolism..

[17] Meilleur KG, Doumatey A, Huang H, Charles B, Chen G, Zhou J (2010). Circulating adiponectin is associated with obesity and serum lipids in west Africans. J Clin Endocrinol Metab..

[18] Yamamoto S, Matsushita Y, Nakagawa T, Hayashi T, Noda M, Mizoue T (2014). Circulating adiponectin levels and risk of type 2 diabetes in the Japanese. Nutr Diab.

[19] Liu Y, Sweeney G (2014). Adiponectin action in skeletal muscle. Best Pract Res Clin Endocrinol Metab.

[20] Liu Y, Chewchuk S, Lavigne C, Brûlé S, Pilon G, Houde V (2009). Functional significance of skeletal muscle adiponectin production, changes in animal models of obesity and diabetes, and regulation by rosiglitazone treatment. Am J Physiol Endocrinol Metab.

[21] Yamauchi T, Kamon J, Ya M, Ito Y, Waki H, Uchida S (2002). Adiponectin stimulates glucose utilization and fatty-acid oxidation by activating AMP-activated protein kinase. Nature medicine.

[22] Fruebis J, Tsao T-S, Javorschi S, Ebbets-Reed D, Erickson MRS, Yen FT (2001). Proteolytic cleavage product of 30-kDa adipocyte complement-related protein increases fatty acid oxidation in muscle and causes weight loss in mice. Proc Natl Acad Sci.

[23] Liu Y, Turdi S, Park T, Morris NJ, Deshaies Y, Xu A (2013). Adiponectin corrects high-fat diet-induced disturbances in muscle metabolomic profile and whole-body glucose homeostasis. Diabetes..

[24] Lindberg S, Jensen JS, Pedersen SH, Galatius S, Frystyk J, Flyvbjerg A, et al. Low adiponectin levels and increased risk of T2DM in patients with myocardial infarction. Diab Care. 2014;37(11):3003–8. https://care.diabetesjournals.org/content/37/11/3003.long. 10.2337/dc14-0932 25078899

[25] Pereira RI, Snell-Bergeon JK, Erickson C, Schauer IE, Bergman BC, Rewers M (2012). Adiponectin dysregulation and insulin resistance in type 1 diabetes. J Clin Endocrinol Metab.

[26] Asayama K, Hayashibe H, Dobashi K, Uchida N, Nakane T, Kodera K (2003). Decrease in serum adiponectin level due to obesity and visceral fat accumulation in children. Obes Res.

[27] Okada-Iwabu M, Yamauchi T, Iwabu M, Honma T, Hamagami K-I, Matsuda K (2013). A small-molecule AdipoR agonist for type 2 diabetes and short life in obesity. Nature.

[28] Holland WL, Scherer PE (2013). Ronning after the adiponectin receptors. Science..

[29] Chalfant CE, Spiegel S (2005). Sphingosine 1-phosphate and ceramide 1-phosphate: expanding roles in cell signaling. J Cell Sci.

[30] Van Brocklyn JR, Williams JB (2012). The control of the balance between ceramide and sphingosine-1-phosphate by sphingosine kinase: oxidative stress and the seesaw of cell survival and death. Comp Biochem Physiol B: Biochem Mol Biol.

[31] Sharma AX, Holland WL. Adiponectin and its hydrolase-activated receptors. J Nat Sci. 2017;3(6):e396. https://pubmed.ncbi.nlm.nih.gov/28758149/. PMC5531184 28758149

[32] Holland WL, Miller RA, Wang ZV, Sun K, Barth BM, Bui HH (2011). Receptor-mediated activation of ceramidase activity initiates the pleiotropic actions of adiponectin. Nat Med.

[33] Patel S, Hoehn K, Lawrence R, Sawbridge L, Talbot N, Tomsig J (2012). Overexpression of the adiponectin receptor AdipoR1 in rat skeletal muscle amplifies local insulin sensitivity. Endocrinology..

[34] Vasiliauskaité-Brooks I, Sounier R, Rochaix P, Bellot G, Fortier M, Hoh F (2017). Structural insights into adiponectin receptors suggest ceramidase activity. Nature..

[35] Danieli-Betto D, Peron S, Germinario E, Zanin M, Sorci G, Franzoso S (2010). Sphingosine 1-phosphate signaling is involved in skeletal muscle regeneration. Am J Phys Cell Phys.

[36] Ieronimakis N, Pantoja M, Hays AL, Dosey TL, Qi J, Fischer KA (2013). Increased sphingosine-1-phosphate improves muscle regeneration in acutely injured mdx mice. Skelet Muscle.

[37] Perreault L, Newsom SA, Strauss A, Kerege A, Kahn DE, Harrison KA (2018). Intracellular localization of diacylglycerols and sphingolipids influences insulin sensitivity and mitochondrial function in human skeletal muscle. JCI insight.

[38] Bruce CR, Risis S, Babb JR, Yang C, Kowalski GM, Selathurai A (2012). Overexpression of sphingosine kinase 1 prevents ceramide accumulation and ameliorates muscle insulin resistance in high-fat diet-fed mice. Diabetes..

[39] Cuvillier O, Pirianov G, Kleuser B, Vanek PG, Coso OA, Gutkind JS (1996). Suppression of ceramide-mediated programmed cell death by sphingosine-1-phosphate. Nature..

[40] Botta A, Liu Y, Wannaiampikul S, Tungtrongchitr R, Dadson K, Park T-S (2019). An adiponectin-S1P axis protects against lipid induced insulin resistance and cardiomyocyte cell death via reduction of oxidative stress. Nutr Metab.

[41] Williamson DF, Parker RA, Kendrick JS (1989). The box plot: a simple visual method to interpret data. Ann Intern Med.

[42] Chen S-Y, Feng Z, Yi X (2017). A general introduction to adjustment for multiple comparisons. J Thorac Dis.

[43] Abdul-Ghani MA, DeFronzo RA. Pathogenesis of insulin resistance in skeletal muscle. J Biomed Biotechnol. 2010;2010. Article ID: 476279. https://www.hindawi.com/journals/bmri/2010/476279/. 10.1155/2010/476279 PMC2860140 20445742

[44] Turcotte LP, Fisher JS (2008). Skeletal muscle insulin resistance: roles of fatty acid metabolism and exercise. Phys Ther.

[45] Patková J, Anděl M, Trnka J (2014). Palmitate-induced cell death and mitochondrial respiratory dysfunction in myoblasts are not prevented by mitochondria-targeted antioxidants. Cell Physiol Biochem.

[46] Yuzefovych L, Wilson G, Rachek L (2010). Different effects of oleate vs. palmitate on mitochondrial function, apoptosis, and insulin signaling in L6 skeletal muscle cells: role of oxidative stress. Am J Physiol Endocrinol Metab.

[47] Hommelberg PPH, Plat J, Sparks LM, Schols AMWJ, van Essen ALM, Kelders MCJM (2011). Palmitate-induced skeletal muscle insulin resistance does not require NF-κB activation. Cell Mol Life Sci.

[48] Sadeghi A, Seyyed Ebrahimi SS, Golestani A, Meshkani R (2017). Resveratrol ameliorates Palmitate-induced inflammation in skeletal muscle cells by attenuating oxidative stress and JNK/NF-κB pathway in a SIRT1-independent mechanism. J Cell Biochem.

[49] Sente T, Van Berendoncks AM, Hoymans VY, Vrints CJ (2016). Adiponectin resistance in skeletal muscle: pathophysiological implications in chronic heart failure. J Cachexia Sarcopenia Muscle.

[50] Mullen KL, Pritchard J, Ritchie I, Snook LA, Chabowski A, Bonen A (2009). Adiponectin resistance precedes the accumulation of skeletal muscle lipids and insulin resistance in high-fat-fed rats. Am J Phys Regul Integr Comp Phys.

[51] Liu Y, Palanivel R, Rai E, Park M, Gabor TV, Scheid MP (2015). Adiponectin stimulates autophagy and reduces oxidative stress to enhance insulin sensitivity during high-fat diet feeding in mice. Diabetes..

[52] Hu W, Bielawski J, Samad F, Merrill AH, Cowart LA (2009). Palmitate increases sphingosine-1-phosphate in C2C12 myotubes via upregulation of sphingosine kinase message and activity. J Lipid Res.

[53] Bandet CL, Tan-Chen S, Bourron O, Le Stunff H, Hajduch E (2019). Sphingolipid metabolism: new insight into Ceramide-induced lipotoxicity in muscle cells. Int J Mol Sci.

[54] McBee ME, Chionh YH, Sharaf ML, Ho P, Cai MW, Dedon PC (2017). Production of superoxide in bacteria is stress-and cell state-dependent: a gating-optimized flow cytometry method that minimizes ROS measurement artifacts with fluorescent dyes. Front Microbiol.

[55] Yang C, Jiang L, Zhang H, Shimoda LA, DeBerardinis RJ, Semenza GL (2014). Analysis of hypoxia-induced metabolic reprogramming. Methods Enzymol.

[56] Ji-Eun K, Seung Eun S, Yong-Woon K, Jong-Yeon K, Sung-Chul P, Yoon-Ki P (2010). Adiponectin inhibits palmitate-induced apoptosis through suppression of reactive oxygen species in endothelial cells: involvement of cAMP/protein kinase a and AMP-activated protein kinase. J Endocrinol.

[57] Zhao W, Wu C, Li S, Chen X (2016). Adiponectin protects palmitic acid induced endothelial inflammation and insulin resistance via regulating ROS/IKKβ pathways. Cytokine..

[58] Japtok L, Schmitz EI, Fayyaz S, Krämer S, Hsu LJ, Kleuser B (2015). Sphingosine 1-phosphate counteracts insulin signaling in pancreatic β-cells via the sphingosine 1-phosphate receptor subtype 2. FASEB J.

[59] Maceyka M, Harikumar KB, Milstien S, Spiegel S (2012). Sphingosine-1-phosphate signaling and its role in disease. Trends Cell Biol.

[60] Frati A, Ricci B, Pierucci F, Nistri S, Bani D, Meacci E (2015). Role of sphingosine kinase/S1P axis in ECM remodeling of cardiac cells elicited by relaxin. Mol Endocrinol.

[61] Kumar A, Byun H-S, Bittman R, Saba JD (2011). The sphingolipid degradation product trans-2-hexadecenal induces cytoskeletal reorganization and apoptosis in a JNK-dependent manner. Cell Signal.

[62] Schumacher F, Neuber C, Finke H, Nieschalke K, Baesler J, Gulbins E, et al. The sphingosine 1-phosphate break-down product (2E)-hexadecenal forms protein adducts and glutathione conjugates in vitro. J Lipid Re. 2017:jlr. M076562. 10.1194/jlr.M076562 PMC5538286 28588048

[63] Borges MC, Lawlor DA, de Oliveira C, White J, Horta BL, Barros AJ (2016). Role of adiponectin in coronary heart disease risk: a Mendelian randomization study. Circ Res.

[64] Ai M, Otokozawa S, Asztalos BF, White CC, Cupples LA, Nakajima K (2011). Adiponectin: an independent risk factor for coronary heart disease in men in the Framingham offspring study. Atherosclerosis..

[65] Sasso FC, Pafundi PC, Marfella R, Calabrò P, Piscione F, Furbatto F (2019). Adiponectin and insulin resistance are related to restenosis and overall new PCI in subjects with normal glucose tolerance: the prospective AIRE study. Cardiovasc Diabetol.

